# Self-assembled molecular nanowires on prepatterned Ge(001) surfaces†

**DOI:** 10.1039/d2sc00490a

**Published:** 2022-04-18

**Authors:** Jing Lyu, Zicong Marvin Wong, Haicheng Sun, Shuo-Wang Yang, Guo Qin Xu

**Affiliations:** Department of Chemistry, National University of Singapore 3 Science Drive 3 117543 Singapore chmxugq@nus.edu.sg; Institute of High Performance Computing, Agency for Science, Technology and Research 1 Fusionopolis Way, #16-16 Connexis 138632 Singapore yangsw@ihpc.a-star.edu.sg

## Abstract

It is a long-standing goal to fabricate conductive molecular nanowires (NWs) on semiconductor surfaces. Anchoring molecules to pre-patterned surface nanostructures is a practical approach to construct molecular NWs on semiconductor surfaces. Previously, well-ordered inorganic Ge NWs were deduced to spontaneously grow onto Pt/Ge(001) surfaces after annealing at an elevated temperature. In this work, we further demonstrate that organic 7,7,8,8-tetracyanoquinodimethane (TCNQ) molecular NWs can self-assemble onto the atomic NWs on Pt/Ge(001) surfaces. The outer nitrogen atoms in TCNQ molecules hybridize with under-coordinated Ge atoms in Ge NWs with an energy release of ∼1.14 eV per molecule, and electrons transfer from Ge NWs to the frontier orbitals of anchored TCNQs resulting in a negatively charged state. This largely tailors the electronic configurations of TCNQs and Pt/Ge(001) surfaces, enhancing the electron transport along the dimer row direction. The TCNQ molecular NWs coupled with the Ge NWs represent an exemplary showcase for the fabrication of molecular NWs on semiconductor surfaces.

## Abstract 2

We reported a strategy for the self-assembly of molecular nanowires (NWs) on Ge(001) surfaces.

## Graphical abstract

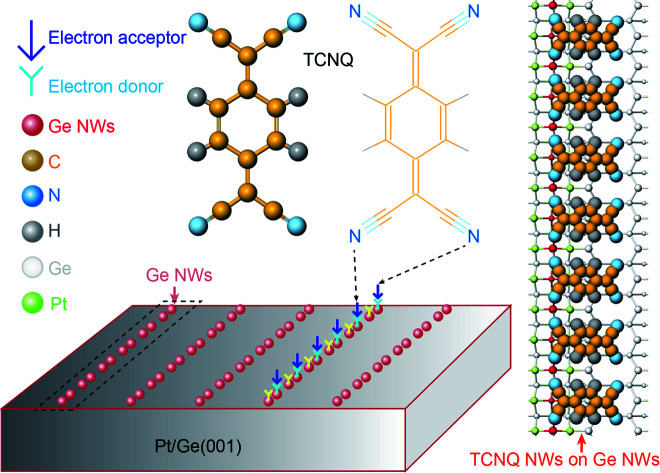

## Introduction

Molecular electronics have been attracting much attention due to the fact that commercially utilized silicon-based electronic devices are approaching their physical limits.^1–3^ Organic molecules can be utilized to design molecular devices.^4–6^ One-dimensional (1D) molecular nanowires (NWs) may serve as conductive channels for molecular devices. Therefore, the fabrication of molecular NWs on semiconductor surfaces becomes important for designing high-performance molecular electronics.

To date, various strategies have been proposed to fabricate molecular NWs on semiconductor substrates.^7^ Among them, self-assembly may be the simplest yet effective approach. For example, the self-assembly of molecular NWs can occur on a predefined position on Si(001) platforms *via* a radical chain reaction mechanism.^8^ Molecules with terminal C≡C, C=C, and C=O groups can easily react with the dangling bonds on silicon surfaces, where the dangling bonds could be created by the removal of H atoms on H-terminated silicon surfaces using an STM tip. These predesigned dangling bonds serve as a starting point for the subsequent radical chain propagation. The reactions involve the breakage of the double or triple bond, thereby resulting in a radical intermediate with a Si–C bond and a C-centred radical. Subsequently, the newly formed radical intermediate is capable of abstracting H atoms from its neighbouring Si atoms, producing a new dangling bond on the nearby Si atom, which continues to interact with another molecule. This series of reactions sets off a chain reaction on hydrogenated silicon surfaces, leading to the growth of molecular NWs on Si(001) and Si(111) surfaces.^8–12^

From the examples stated above, we notice that dangling bonds play critical roles in the self-assembly of molecular NWs on semiconductor surfaces. The presence of dangling bonds is a prerequisite to fabricate covalently adsorbed molecular NWs onto semiconductor surfaces. It was reported that atomic NWs, extending for hundreds of nanometres, can be self-organized on Pt/Ge(001) surfaces.^13^ These NWs are constructed of under-coordinated Ge atoms, possibly suggesting the presence of dangling bonds on NWs. In this regard, they are expected to be good platforms for fabricating molecular NWs, because the dangling bonds on Ge NWs on Pt/Ge(001) surfaces can serve as potential points for the assembly of molecular NWs. Moreover, an appropriate space should be provided to accommodate the molecular NWs. On the Pt/Ge(001) platform, Ge NWs are spaced by either 1.6 nm or 2.4 nm. In our experimental observations, we indeed found that 7,7,8,8-tetracyanoquinodimethane (TCNQ) molecular NWs can be self-organized on Ge NWs on Pt/Ge(001) surfaces. This observation of TCNQ molecular NWs is an exemplary showcase for the fabrication of molecular NWs on semiconducting surfaces.

## Methods

Low-temperature scanning tunnelling microscopy (LT-STM) characterization was carried out in a custom-designed Unisoku LT-STM system with the base pressure maintained in a low 10^−10^ Torr range at 77 K, using the constant current mode with a commercial Pt–Ir tip. The bias voltages were applied to the sample. Ge(001) was cut from one-side-polished n-type wafers (commercially available from AXT Inc.). Samples were ultrasonically cleaned in propanol for 15 min several times and then dried under nitrogen gas. Subsequently, the prepared sample was fixed onto a Mo sample holder. Pristine Ge(001) surfaces with c(4 × 2) and p(2× 2) periodicities were prepared through several cycles of 500 eV Ar^+^ ion sputtering and annealing at around 1100 K. Pt atoms were then resistively evaporated onto dimerized Ge(001) surfaces at about 873 K for 10 min. TCNQ molecules (98%, Aldrich) were deposited on Pt/Ge(001) surfaces at 300 K using a Knudsen cell at 393 K. The calculation details are presented in the ESI.†^14–25^

## Results and discussion

**Fig. 1:**
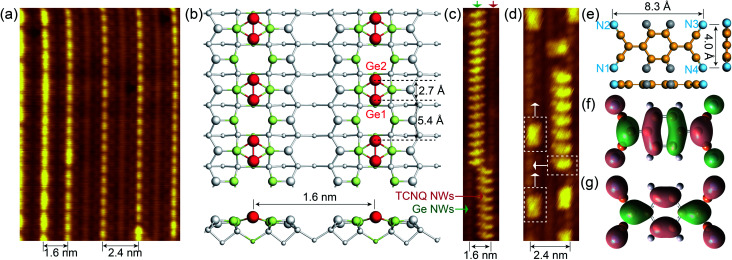
The self-assembly of TCNQ molecular NWs along Ge NWs on Pt/Ge(001) surfaces. (a) Filled-state STM images of Ge NWs self-organized on Pt/Ge(001) surfaces (*V*_s_ = −1.5 V; *I*_t_ = 100 pA; *T* = 77 K). (b) Top and side views of the atomic configuration of Ge NWs on Pt/Ge(001) surfaces, and the 4 × 2 supercell is shown in Fig. S1a and b.† The white and light green spheres stand for Ge and Pt atoms, respectively. The larger spheres are higher than other atoms. The red spheres represent the Ge NWs, and the Ge NWs are constructed of Ge1–Ge2 dimers. (c and d) Filled-state STM topographies of TCNQ molecular NWs propagating along the Ge NWs on Pt/Ge(001) surfaces. (e) Top and side views of an isolated TCNQ molecule. The grey, yellow, and blue spheres represent H, C, and N atoms, respectively. (f) LUMO and (g) HOMO orbitals of a TCNQ molecule calculated with B3LYP/6-31G*.

Noble metals (*i.e.*, Au and Pt) are known to trigger the self-assembly of atomic NWs on Ge(001) surfaces.^13,26–29^ DFT calculations revealed that self-organized NWs on Pt/Ge(001) surfaces are constituted by under-coordinated Ge atoms (Fig. 1b, S1a and b†), suggesting the presence of dangling bonds on the NWs.^30^ As aforementioned, dangling bonds on semiconductor surfaces can capture molecules by chemically bonding with them, and then setting off the self-assembly of molecular NWs on the semiconductor surfaces. TCNQ molecules, as archetypal electron acceptors, are widely utilized to prepare charge transfer complexes, showing potential in engineering molecular electronics.^31–34^ The electronic properties of TCNQ are mainly determined by the population of the lowest unoccupied molecular orbital (LUMO) (Fig. 1f). Here, the cyano groups in TCNQ are expected to chemically react with under-coordinated Ge atoms in the NWs on Pt/Ge(001) surfaces, abstracting electrons from the Ge atoms in the NWs. Moreover, Fig. 1e indicates that TCNQ is a planar molecule with a width of 4.0 Å and a length of 8.3 Å. The Ge NWs in Fig. 1a are spaced by 1.6 or 2.4 nm, providing adequate room to accommodate TCNQs. The Ge dimers in the NWs are spaced by 5.4 Å, further suggesting that the outer nitrogen atoms, separated by 4.0 Å (*d*_N1–N2_ = *d*_N3–N4_ =4.0 Å), in a TCNQ molecule possibly react with Ge atoms. As expected, TCNQ molecular NWs were experimentally observed alongside the Ge NWs on Pt/Ge(001) surfaces (see Fig. 1c and d). Furthermore, individual TCNQ molecules can adsorb in another orientation, marked with white dotted rectangles in Fig. 1d, perpendicular to the TCNQ molecular NWs.

**Fig. 2:**
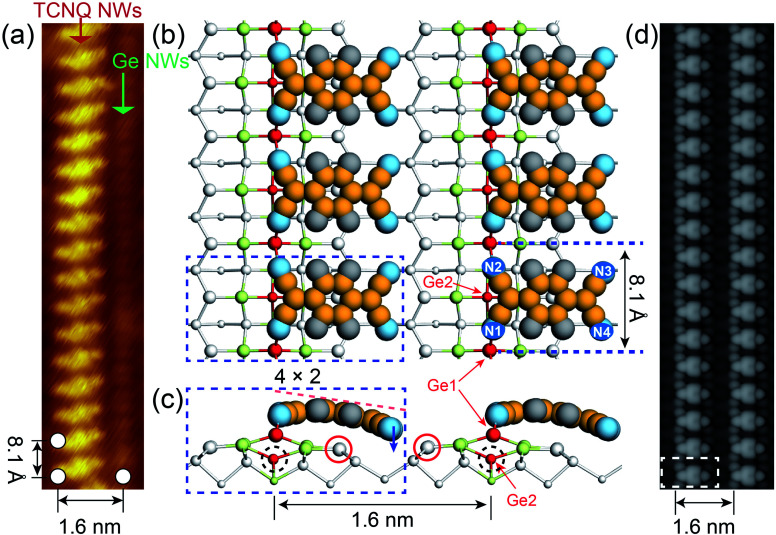
The self-assembled TCNQ molecular NWs onto Ge NWs on Pt/Ge(001) surfaces. (a) Filled-state STM images of self-assembled TCNQ molecular NWs on Pt/Ge(001) surfaces (*V*_s_ = −1.5 V; *I*_t_ = 100 pA; *T* = 77 K). Top (b) and side (c) views of the DFT-optimized configurations of TCNQ molecular NWs on Pt/Ge(001) surfaces. The dotted rectangles indicate the structural unit for DFT calculations. The white, light green, yellow, grey, and blue balls represent Ge, Pt, C, H, and N atoms, respectively. The larger spheres are higher than other atoms. The chemisorption of TCNQ causes the rupture of the Ge–Ge dimer (*i.e.*, Ge1 and Ge2) in Ge NWs; Ge1 forms covalent bonds with two cyano groups on the left side of the TCNQ, and Ge2 moves downwards to react with Pt atoms in the bottom. (d) Simulated filled-state STM images of the TCNQ molecular NWs on Pt/Ge(001) surfaces (*V*_s_ = −1.5 V).

Fig. 2a shows that the TCNQs anchor onto the Ge NWs with their cyano groups located above the Ge NWs, generating a flat adsorption geometry with respect to the Pt/Ge(001) surface, and Ge NWs could be observed adjacently about 1.6 nm away. DFT calculations indicate that TCNQ molecules prefer to chemically bond to the Ge NWs instead of physisorption. The calculation results in Fig. 2b suggest that the nitrogen atoms on the left side of the TCNQ (*i.e.*, N1 and N2) form covalent bonds with the Ge1 atoms in NWs, and the remaining nitrogen atoms (*i.e.*, N3 and N4) on the right side (see the blue arrow in Fig. 2c) are situated in the trench between two Ge NWs. The formation of Ge–N bonds causes the rupture of the Ge1–Ge2 dimer in Ge NWs. The Ge1 atom chemically interacts with the N1 and N2 atoms, and the Ge2 atom, marked with dotted circles, moves downwards to react with Pt atoms. No covalent bond is formed between the Ge2 atom and TCNQ molecule. Furthermore, the N3 and N4 atoms, indicated with a blue arrow, slightly move downwards due to the presence of dangling bonds of the Ge atoms (red circles in Fig. 2c) near Pt atoms. The conjugated π-system extending across the TCNQ makes it a planar and rigid molecule in the gas-phase state. The adsorbed TCNQ becomes flexible if electrons are transferred to the cyano groups since the peripheral carbon atoms of TCNQ turn into the nonplanar sp^3^ hybridization.^35^ Moreover, the LUMO of a TCNQ is constructed of antibonding π orbitals gathering around the double bonds in TCNQ; therefore, the electron filling of the LUMO can weaken these double bonds and make TCNQ flexible.^35^ The negatively charged TCNQ molecular NWs are no longer rigidly planar, accounting for the bending of anchored TCNQs.

The chemical adsorption of TCNQs onto the Ge NWs on Pt/Ge(001) surfaces is an exothermic reaction with an energy release of 1.14 eV per molecule. Energy variation during this process is calculated using the formula: *E*_ads_ = *E*[TCNQ/Ge(001)] − *E*(TCNQ) − *E*[Ge(001)], where *E*[TCNQ/Ge(001)], *E*(TCNQ) and *E*[Ge(001)] are the energies of the anchored TCNQ on Pt/Ge(001) surfaces, the isolated TCNQ molecule, and the Pt/Ge(001) surfaces, respectively.

The DFT-simulated occupied state STM in Fig. 2d presents well-ordered TCNQ molecular NWs propagating along the Ge NWs on Pt/Ge(001) surfaces. Two TCNQs are 8.1 Å apart alongside NWs, double the distance of the neighbour Ge dimers on Ge(001)c(4 × 2) surfaces, which is consistent with the experimental value of ∼8.1 Å (Fig. 2a). As aforementioned, the TCNQ chemisorption leads to the Ge atom rearrangement in Ge NWs. Given that the Ge NWs near the TCNQ NWs (see Fig. 2a) are not fully occupied in the experiment, the slight rotation of anchored TCNQs in experiment may be attributed to the effect from the nearest neighbour Ge NWs. Moreover, a single TCNQ can be adsorbed on either side of Ge NWs; however, TNCQs in Fig. 2a appear on the same side. The nearest distance between two TCNQs is about 3.5 Å, suggesting a pronounced van der Waals interaction responsible for the low entropy configuration.

**Fig. 3:**
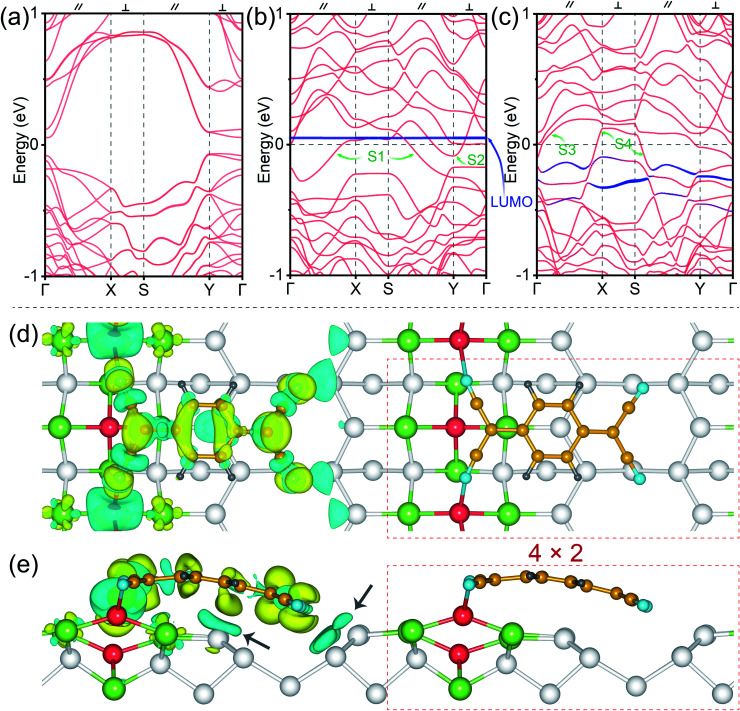
The electronic band structures of (a) dimerized Ge(001) surfaces, (b) Pt/Ge(001) surfaces with isolated TCNQs and (c) anchored TCNQs chemically bonded with Ge NWs. The navy blue lines represent the bands contributed by TCNQ molecules. The thickness of the lines is proportional to their weight of contribution. The Fermi level (*E*_F_) is adjusted to 0 eV. The charge density difference for the anchored TCNQs on Pt/Ge(001) surfaces is presented by the isosurface of 0.002 e Bohr^−3^ in (d) top and (e) side views, where yellow and cyan colours refer to charge accumulation and depletion, respectively. The white, light green, yellow, grey, and blue balls represent Ge, Pt, C, H, and N atoms, respectively. The dotted rectangles indicate the structural unit without showing the charge transfer difference.

To further investigate the charge transfer between the TCNQ molecular NWs and Ge NWs, the band structures and charge density differences are illustrated in Fig. 3. Fig. 3a shows the band structure of the dimerized Ge(001) surface (see the atomic structure in Fig. S1e and f†), and this surface is semiconducting with a bandgap around the Fermi level, *E*_F_. The red bands in Fig. 3b refer to the Pt/Ge(001) substrates, and the navy blue energy levels correspond to isolated TCNQs (see the atomic structure in Fig. S1c and d†). One can note that the Pt modified Ge(001) surfaces behave as a metal since the S1 and S2 bands pass through the Fermi level (Fig. 3b), and the energy band dispersions perpendicular to the Ge dimer row direction are weaker than the bands parallel to the Ge dimer row direction, indicating that the NWs are more conducting. Moreover, compared to S1 and S2, the bands S3 and S4 across the Fermi level are more dispersed around the Fermi level after being chemically bonded with TCNQ molecular NWs, as displayed in Fig. 3c. More dispersive bands typically suggest smaller effective masses and higher carrier mobility; therefore, the conductivity along the NW direction is enhanced due to the presence of the TCNQ molecular NWs. Additionally, no band passes through the Fermi level along the X–S and Y–Γ paths perpendicular to the direction of NWs, possibly suggesting the suppression of conductivity perpendicular to the TCNQ molecular NWs. Moreover, the TCNQ molecular energy level in Fig. 3b is isolated without the chemical interactions between TCNQs and the Pt/Ge(001) surfaces (see the structural model in Fig. S1c and d†). The molecular energy level of TCNQ in close vicinity to the Fermi level is considered as the LUMO. It is mentioned in Fig. 2b that the Ge dimers of NWs on the Pt/Ge(001) surfaces are rearranged after the formation of TCNQ molecular NWs. Therefore, compared to Fig. 3b, the electronic band structures of TCNQ molecular NWs (Fig. 3c) are largely modified. The pronounced dispersions of the HOMO and LUMO of TCNQs reflect hybridization between TCNQ and Pt/Ge(001) surfaces (Fig. S2†). In particular, the LUMO of TCNQ presents a concentrated distribution from 0.1 to 0.5 eV (Fig. 3c) below the Fermi level after chemically reacting with Ge NWs on Pt/Ge(001) surfaces, suggesting substantial electron charge transfer from Ge NWs to TCNQs. This charge transfer is further visualized in Fig. 3d. The Bader charge analysis suggests that each Ge in Ge–N bonds donates about 0.6 electrons to the anchored TCNQ. Notably, the charge transfer occurs between Ge NWs and TCNQs and between the Ge atoms near the Pt arrays and TCNQs indicated by the black arrows in Fig. 3e. The electron transfer of the Ge atoms near the Pt arrays should be partially responsible for the bending of anchored TCNQs on Ge NWs because of the Coulomb interactions. In general, the Bader charge analysis indicates the transfer of a total of 1.1 electrons from the substrate to each anchored TCNQ. A closer inspection of the whole charge transfer pattern in Fig. 3d indicates that the electron accumulated region resembles the LUMO of TCNQs (Fig. 1f). This further confirms the electron transfer to the LUMO of TCNQs, generating negatively charged TCNQ molecular NWs. To sum up, the adsorption of TCNQ molecular NWs causes pronounced alterations in the electronic and geometrical configurations of both TCNQs and Pt/Ge(001) surfaces.

## Conclusions

In this work, the deposition of TCNQs onto Ge NWs on Pt/Ge(001) surfaces at room temperature results in the self-assembly of TCNQ molecular NWs. The Ge NWs can serve as fences on Pt/Ge(001) surfaces, providing adequate space for the growth of TCNQ molecular NWs. The cyano groups in TCNQs can chemically react with the under-coordinated Ge NWs, generating nearly flat TCNQ molecular NWs along the Ge NWs. Electrons transfer from the Ge NWs to the TCNQs, making the TNCQ molecular NWs negatively charged. Our work not only provides an effective approach for fabricating molecular NWs on prepatterned semiconductor surfaces but also offers important insights into tailoring the surface properties of semiconductor surfaces.

## Data availability

The data supporting the findings of this study are available within in the article and in the ESI.† Raw data are available from the corresponding author upon reasonable request.

## Author contributions

Jing Lyu: writing – original draft, data curation, formal analysis, investigation; Zicong Marvin Wong: writing – review & editing; Haicheng Sun: resources; Shuo-Wang Yang: supervision, writing – review & editing; Guo Qin Xu: supervision, writing – review & editing.

## Conflicts of interest

There are no conflicts to declare.

## Supplementary Material

SC-013-D2SC00490A-s001
