# Supplementary material for: Self-assembled molecular nanowires on prepatterned Ge(001) surfaces
Source: Chem Sci. 2022 Apr 18;13(19):5674–9. doi: 10.1039/d2sc00490a (PMC9116370; doi:10.1039/d2sc00490a)
Supplement: SC-013-D2SC00490A-s001 [file SC-013-D2SC00490A-s001.pdf]

Electronic Supplementary Material (ESI) for Chemical Science.
This journal is © The Royal Society of Chemistry 2022

Supporting Information

Self-Assembled Molecular Nanowires on Prepatterned Ge(001) Surfaces

Jing Lyu[a], Zicong Marvin Wong[b], Haicheng Sun[a], Shuo-Wang Yang*[b], and Guo Qin Xu*[a]

[a]*Department of Chemistry, National University of Singapore, 3 Science Drive 3, 117543, Singapore*

[b]*Institute of High Performance Computing, Agency for Science, Technology and Research, 1 Fusionopolis Way, # 16-16 Connexis, 138632, Singapore*

*Corresponding Author. E-mail: yangsw@ihpc.a-star.edu.sg; chmxugq@nus.edu.sg

A. Computational methods

DFT calculations were carried out with the Perdew, Burke, and Ernzerhof (PBE)[1] exchange-correlation based on the generalized gradient approximation (GGA)[2,3] as implemented in the framework of Vienna *ab initio* Simulation Package (VASP)[4,5]. The projector augmented wave (PAW)[6,7] was utilized to describe the electron-ion interactions and plane wave cutoffs were set to 500 eV. The Ge(001) surface was built with ten layers of Ge atoms and a vacuum region of more than 15 Å. Five of ten atomic layers were allowed to relax and the remaining layers were fixed at the bulk structure. One layer of hydrogen atoms was introduced to passivate the bottom Ge surface atoms. A 4 × 2 supercell with 6 × 12 Monkhorst-Pack (MP) *k*-point meshes was built to model the Ge NWs on Pt/Ge(001) surfaces.[8] The atomic coordinates were optimized until the Hellmann-Feynman forces were smaller than 0.02 eV/Å. The van der Waals (vdW) correlation by the DFT-D3 method was taken into account in all calculations.[9] VASPKIT, a pre-and post-processing tool for VASP code, was utilized to prepare some input files.[10] Calculated STM images were obtained using the Tersoff-Hamann method[11]: the tunneling current is proportional to the local density of states integrated from bias to Fermi level, and were visualized using the p4VASP program (see http://www.p4vasp.at/). The molecular orbitals were calculated *via* Gaussian16 with B3LYP (Becke, 3-parameter, Lee-Yang-Parr) and 6-31G* basis set.[12]

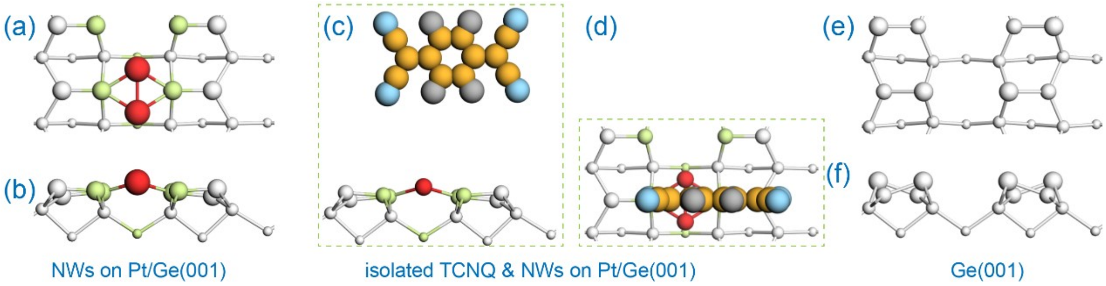

Fig. S1 Top and side views of the structural models with the 4 × 2 supercell for NWs on Pt/Ge(001), Pt/Ge(001) surfaces with isolated TCNQ, and dimerized Ge(001) surfaces. The white, light green, yellow, grey, and blue balls represent Ge, Pt, C, H, and N atoms, respectively. The larger spheres are higher than other atoms.

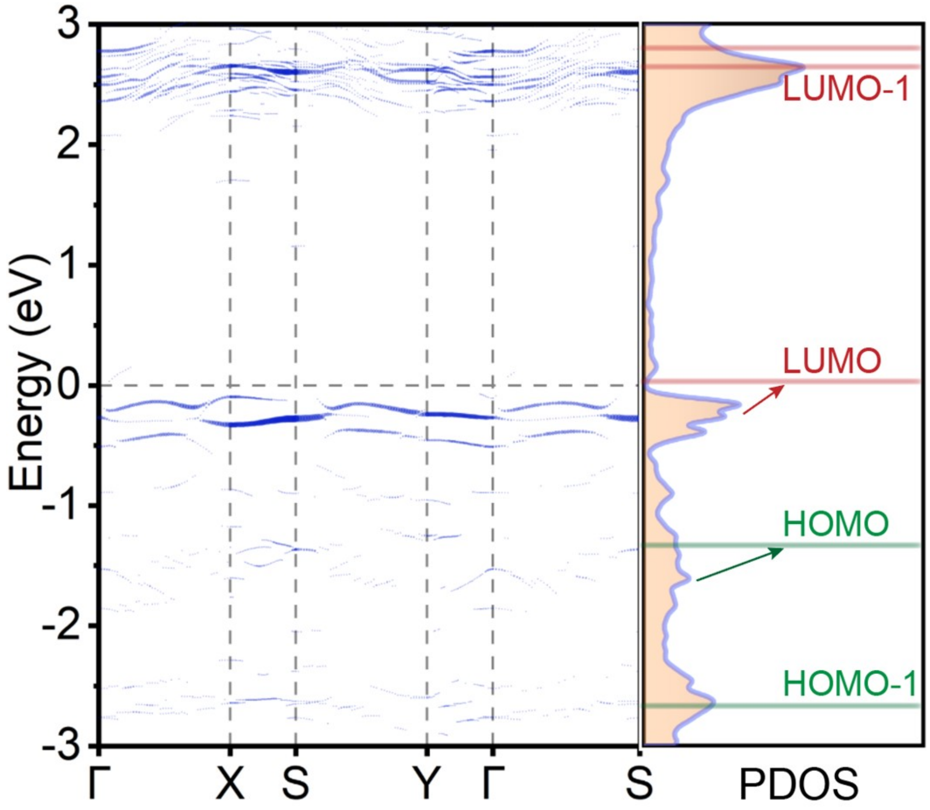

Fig. S2 The electronic band structures of the Pt/Ge(001) surfaces with TCNQ molecular NWs and their corresponding projected density-of-states (PDOS). The thickness of the lines is proportional to their weight of contribution. The Fermi level (E_F) is adjusted to 0 eV. The HOMO and LUMO of the gas-phase TCNQ molecule are marked by the green and red lines, respectively.

References

1 W. Kohn and L. J. Sham, *Phys. Rev.*, 1965, **140**, A1133-A1138.

2 J. P. Perdew, K. Burke and Y. Wang, *Phys. Rev. B*, 1996, **54**, 16533.

3 J. P. Perdew, K. Burke and M. Ernzerhof, *Phys. Rev. Lett.*, 1996, **77**, 3865-3868.

4 J. F. G. Kresse, *Phys. Rev. B*, 1996, **54**, 11169-11186.

5 J. F. G. Kresse, *Comput. Mater. Sci.*, 1996, **6**, 15-50.

6 D. J. G. Kresse, *Phys. Rev. B*, 1999, **59**, 1758-1775.

7 P. E. Blöchl, *Phys. Rev. B*, 1994, **50**, 17953-17979.

8 H. J. Monkhorst and J. D. Pack, *Phys. Rev. B*, 1976, **13**, 5188-5192.

9 S. Grimme, S. Ehrlich and L. Goerigk, *J. Comput. Chem.*, 2011, **32**, 1456-1465.

10 V. Wang, N. Xu, J.-C. Liu, G. Tang and W.-T. Geng, *Comput. Phys. Commun.*, 2021, 108033.

11 J. Tersoff and D. R. Hamann, *Phys. Rev. B*, 1985, **31**, 805.

12 M. J. Frisch, G. W. Trucks, H. B. Schlegel, G. E. Scuseria, M. A. Robb, J. R. Cheeseman, G. Scalmani, V. Barone, G. A. Petersson, H. Nakatsuji, X. Li, M. Caricato, A. V. Marenich, J. Bloino, B. G. Janesko, R. Gomperts, B. Mennucci, H. P. Hratchian, J. V. Ortiz, A. F. Izmaylov, J. L. Sonnenberg, D. Williams-Young, F. Ding, F. Lipparini, F. Egidi, J. Goings, B. Peng, A. Petrone, T. Henderson, D. Ranasinghe, V. G. Zakrzewski, J. Gao, N. Rega, G. Zheng, W. Liang, M. Hada, M. Ehara, K. Toyota, R. Fukuda, J. Hasegawa, M. Ishida, T. Nakajima, Y. Honda, O. Kitao, H. Nakai, T. Vreven, K. Throssell, J. A. Montgomery Jr, J. E. Peralta, F. Ogliaro, M. J. Bearpark, J. J. Heyd, E. N. Brothers, K. N. Kudin, V. N. Staroverov, T. A. Keith, R. Kobayashi, J. Normand, K. Raghavachari, A. P. Rendell, J. C. Burant, S. S. Iyengar, J. Tomasi, M. Cossi, J. M. Millam, M. Klene, C. Adamo, R. Cammi, J. W. Ochterski, R. L. Martin, K. Morokuma, O. Farkas, J. B. Foresman and D. J. Fox, *Gaussian 16, Revision A.03*, Gaussian, Inc., Wallingford CT, 2016.
